# Eculizumab in gemcitabine-induced thrombotic microangiopathy: experience of the French thrombotic microangiopathies reference centre

**DOI:** 10.1186/s12882-021-02470-3

**Published:** 2021-07-21

**Authors:** Maximilien Grall, Florence Daviet, Noémie Jourde Chiche, François Provot, Claire Presne, Jean-Philippe Coindre, Claire Pouteil-Noble, Alexandre Karras, Dominique Guerrot, Arnaud François, Ygal Benhamou, Agnès Veyradier, Véronique Frémeaux-Bacchi, Paul Coppo, Steven Grangé

**Affiliations:** 1 Medical Intensive Care Unit, Rouen University Hospital, 37 boulevard Gambetta, 76031 Rouen Cedex, France; 2 French TMA Reference Centre, Hopital Saint-Antoine, Sorbonne Université, AP-HP, Paris, France; 3 Department of Nephrology, Conception University Hospital, APHM, Marseille, France; 4 Department of Nephrology, Lille University Hospital, Lille, France; 5 Department of Nephrology, Amiens University Hospital, Amiens, France; 6 Department of Nephrology, Le Mans General Hospital, Le Mans, France; 7 Department of Nephrology, E. Herriot Hospital, Lyon I university, Lyon, France; 8 Department of Nephrology, Georges Pompidou Hospital, APHP, Paris, France; 9 Department of Nephrology, Rouen University Hospital, Rouen, France; 10 Department of Pathology, Rouen University Hospital, Rouen, France; 11 Department of Internal Medicine, Rouen University Hospital, Rouen, France; 12 Department of Biological Hematology, Lariboisière University Hospital, APHP, Paris, France; 13 Department of immunology, Georges Pompidou Hospital, APHP, Paris, France; 14 Department of Hematology, Hopital Saint-Antoine, Sorbonne Université, AP-HP, Paris, France

**Keywords:** Coagulation, thrombotic disorders and therapies, Cancer and thrombosis, Eculizumab, Gemcitabine-induced thrombotic microangiopathy

## Abstract

**Background:**

Gemcitabine is a broadly prescribed chemotherapy, the use of which can be limited by renal adverse events, including thrombotic microangiopathy (TMA).

**Methods:**

This study evaluated the efficacy of eculizumab, a monoclonal antibody targeting the terminal complement pathway, in patients with gemcitabine-induced TMA (G-TMA). We conducted an observational, retrospective, multicenter study in 5 French centres, between 2011 and 2016.

**Results:**

Twelve patients with a G-TMA treated by eculizumab were included. The main characteristics were acute renal failure (100%), including stage 3 acute kidney injury (AKI, 58%) and renal replacement therapy (17%), hypertension (92%) and diffuse oedema (83%). Eculizumab was started after a median of 15 days (range 4–44) following TMA diagnosis. A median of 4 injections of eculizumab was performed (range 2–22). Complete hematological remission was achieved in 10 patients (83%) and blood transfusion significantly decreased after only one injection of eculizumab (median of 3 packed red blood cells (range 0–10) before treatment vs 0 (range 0–1) after one injection, *P* < 0.001). Two patients recovered completely renal function (17%), and 8 achieved a partial remission (67%). Compared to a control group of G-TMA without use of eculizumab, renal outcome was more favourable. At the end of the follow up, median eGFR was 45 vs 33 ml/min/1.73m^2^ respectively in the eculizumab group and in the control group.

**Conclusions:**

These results suggest that eculizumab is efficient on haemolysis and reduces transfusion requirement in G-TMA. Moreover, eculizumab may improve renal function recovery.

**Supplementary Information:**

The online version contains supplementary material available at 10.1186/s12882-021-02470-3.

## Key points

- In G-TMA, eculizumab is efficient in controlling the hematological disorders and may improve renal function recovery
- C5b9 deposits in kidney biopsies suggest the role of complement activation in gemcitabine-induced TMA

## Background

Thrombotic microangiopathy (TMA) syndromes are characterized by a microangiopathic hemolytic anemia, peripheral thrombocytopenia, and organ injury of variable severity [1]. The principal subtypes of TMA are thrombotic thrombocytopenic purpura (TTP) mainly due to anti-ADAMTS13 autoantibodies and the hemolytic uremic syndrome (HUS) associated with shigatoxin-related endothelial toxicity (shiga-toxin related HUS) or with complement alternative pathway dysregulation (atypical HUS or aHUS). TMA may also result from drug exposure, the most usual agents being calcineurin inhibitors, quinine, antiplatelet agents as well as antineoplastic agents [2]. Gemcitabine is a pyrimidine antimetabolite used for the treatment of a wide range of malignancies. The reported incidence of gemcitabine-induced TMA (G-TMA) in the literature was initially low (0.015%) [3] but but a rising number of cases have since been documented with the increasing use of gemcitabine [4–7].

Beyond permanent discontinuation of gemcitabine and supportive care, the optimal management of G-TMA is not well codified [5, 8]. As opposed to TTP, G-TMA generally responds poorly to therapeutic plasma exchange (TPE) and prognosis is dismal [9]. Although there is no complement alternative pathway-related abnormalities described, the severe renal injury and normal ADAMTS13 are reminiscent of HUS, in which complement blockade is remarkably efficient [10]. Single reports suggested the efficacy of eculizumab in G-TMA [11], a monoclonal antibody directed against the complement protein C5 that has been approved for treatment of atypical HUS. In this context, the present study evaluated the efficacy of eculizumab in a retrospective series of patients with G-TMA.

## Methods

### Study design

We conducted an observational, retrospective, multicenter study including all patients with G-TMA treated by eculizumab in 5 French centres, between 2011 and 2016.

### Patients

Patients who were included in the study met the following criteria: evidence of microangiopathic hemolytic anemia, including schistocytes on peripheral blood smear, thrombocytopenia (< 150 G/L), increased lactate dehydrogenase levels (> Upper limit of normal), low serum haptoglobin < normal and/or renal TMA proven by kidney biopsy. Only one of these criteria could be missing. The diagnosis was retained by the team which took charge of the patient with discontinuation of treatment with gemcitabine. Patients with a TMA attributed to an uncontrolled cancer, as defined by erythroblastosis, metastatic bone marrow infiltration, impaired general condition, and low-cumulative dose gemcitabine (< 5000 mg/m^2^) were excluded [11–13]. Patients treated with another chemotherapy concomitantly with gemcitabine were excluded. Patients with a positive shiga-toxin or ADAMTS13 activity < 10% were also excluded.

Patients were treated with eculizumab according to the regimen previously reported [14]. It consisted generally in 4 weekly infusions 900 mg IV. In responders, a maintenance treatment was started every 2 weeks at week 5, 1200 mg. The number of infusions was left at the discretion of the practitioner.

Hematological and renal responses were evaluated, based on data that were systematically extracted from the clinical record. Hematological response was defined by normalization of hematologic values (a normal platelet count and lactate dehydrogenase level) as previously described [14]. The transfusion needs were calculated over a period going from the admission of the patient to the end of the treatment with eculizumab. Renal response was considered as complete if serum creatinine level returned to baseline and as partial if serum creatinine level decreased by 15% or more.

Acute renal injury (AKI) was assessed according to KDIGO classification 2012. To make possible the comparison between the two groups, we chose to use the CKD-EPI formula for the estimation of the eGFR (glomerular filtration rate), despite we are aware of the limits in the context of AKI. The eGFR of dialysed patients was estimated at 0 ml/min.

We compared patients with G-TMA treated with eculizumab with a control cohort of patients who did not receive eculizumab treatment. Using the French national network, 14 patients were selected using criteria of G-TMA described above without eculizumab therapy. Patients were matched by age and baseline renal function.

This study was approved by the institutional review board of Rouen University Hospital in accordance with the Declaration of Helsinki, and the French Data Protection Authority (“Commission Nationale Informatique et Libertés,” CNIL, authorization n°911,539, and “Comité consultatif sur le traitement de l’information en matière de recherche dans le domaine de la santé,” CCTIRS, authorization n°11.537, Paris, France).

### Statistical methods

Median with range and percentage (%) were respectively determined for continuous and categorical variables. Differences between groups were assessed by the chi-square test or Fisher’s exact test for categorical variables and by the Mann–Whitney U test for continuous variables [15].

## Results

Twelve patients with a G-TMA treated by eculizumab were included (10 women, 2 men). None had a past history of chronic renal failure. Gemcitabine was prescribed for ovarian (*n* = 5, 41.7%), pancreatic (*n* = 4, 33.3%), pulmonary (*n* = 2, 16.7%) or uterine (*n* = 1, 8.3%) cancer. TMA occurred after a median of 6 months (range 1.7–16) after initiation of gemcitabine and a median cumulative dose of 31.2 g (range, 9.0–48.0) (Table 1). The main characteristics were microangiopathic hemolytic anemia (100%), thrombocytopenia (92%), acute renal failure (100%), including stage 3 acute kidney injury (AKI, 58%), and renal replacement therapy (17%), hypertension (92%) and diffuse oedema (83%). The median maximum serum creatinine level was 21 mg/l (range, 10–76). Quantitative analysis of the complement alternative pathway (CFH, CFI, C3, C1 inhibitor, CD46/MCP and anti-factor H antibodies) was available in 9 patients (75%), and revealed no factor deficiency (Supplemental data). Screening for genetic mutation was performed for one patient and was negative (Genes assessed were factor H, factor I, factor B, MCP, C3 and thrombomodulin). Bone marrow aspiration was realized in 4 patients with no evidence of metastatic infiltration.

**Table 1:** Clinical features of patients in the eculizumab group

Patient	Age (years old)	Type of cancer	Cumulative dose of gemcitabine (mg)	Time to eculizumab initiation (days) after TMA / Number of injection	Staging of AKI	Hemoglobin level (g/dl)	Platelets count (G/l)	LDH ratio (x normal)	Serum Creatinine level at diagnosis (mg/l)	Hematological response	Renal response	Reduction of creatinine level at the end of the follow-up (mg/l)	Outcome / Time to death or time of last follow-up for still alive patients (months)
1	36	Ovarian, M+	23,760	7 d / 4	2	9.3	76	1.8	18.0	Yes	Partial	2.9	Deceased / 9 m
2	64	Ovarian, M+	16,300	7 d / 3	3, RRT	6.9	23	2.5	28.0	No	No	0	Deceased / 1 m
3	54	Pancreatic, M+	31,000	44 d / 22	2	8.5	27	2.2	14.3	Yes	Partial	2.5	Alive / 47 m
4	69	Pancreatic, M+	9040	27 d / 3	3, RRT	7.3	11	2.4	18.0	No	No	0	Deceased / 2 m
5	64	Ovarian, NA	48,000	13 d / 7	2	9.7	130	2.2	22.5	Yes	Partial	7.1	Deceased / 13 m
6	68	Pancreatic, M-	30,000	34 d / 14	3	7.9	13	4.6	17.1	Yes	Complete	10.6	Deceased / 10 m
7	59	Pulmonary, M+	31,200	6 d / 5	3	11.0	137	1.5	31	Yes	Partial	6.9	Alive / 14 m
8	57	Pulmonary, M+	42,500	26 d / 4	3	7.6	150	1.5	76	Yes	Partial	16	Deceased / 3 m
9	52	Uterine, M+	47,000	4 d / 2	3	8.7	48	2.2	70	Yes	Partial	47	Alive / 5 m
10	50	Pancreatic, M+	15,000	19 d / 2	1	8.0	139	2.2	10.2	Yes	Complete	3	Alive / 5 m
11	56	Ovarian, M+	38,000	18 d / 3	3	7.3	144	3.7	24	Yes	Partial	10	Alive / 4 m
12	55	Ovarian, M+	32,000	7 d / 4	3	8.1	122	1.8	64	Yes	Partial	29	Alive / 6 m

*TMA* Thrombotic microangiopathy, *AKI* Acute kidney injury (AKI was assessed according to KDIGO classification 2012), *LDH* Lactate, dehydrogenase, *RRT* Renal replacement therapy, *M-* No metastatic, *M+* Metastatic, *NA* Data not available

**Fig. 1:**
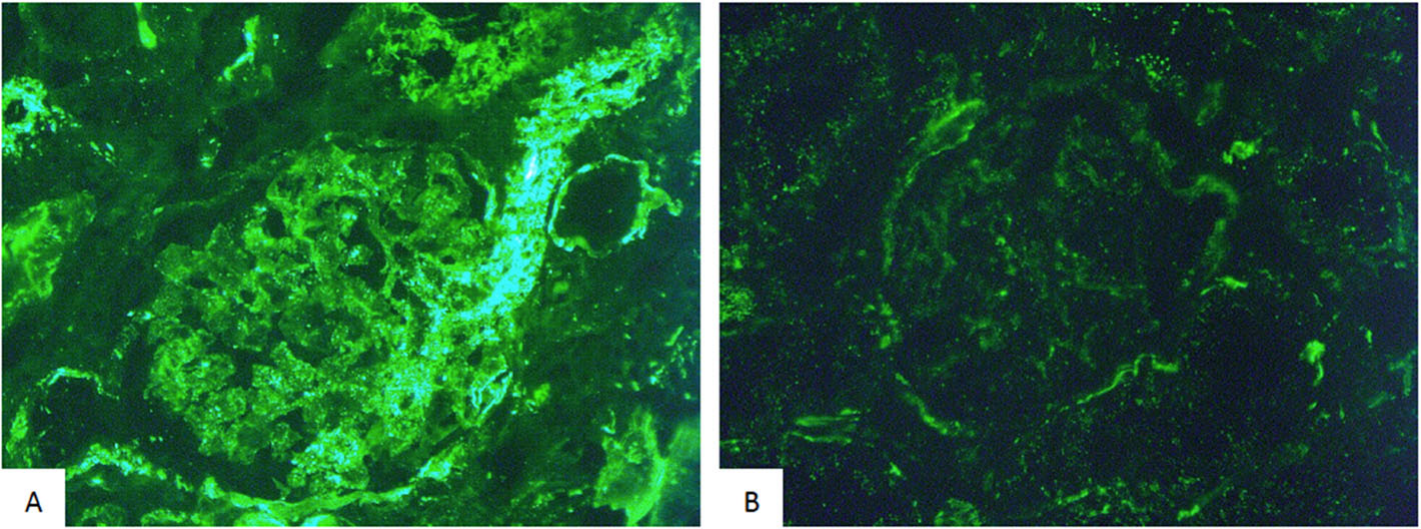
Kidney biopsy in gemcitabine-induced TMA. By immunofluorescence, kidney biopsy of G-TMA patients (**A**) showed deposits of membrane attack complex C5b9 in the glomerular and tubular membrane and also in the capillary walls, as compared to control patients with glomerular disease (minimal change disease) (**B**)

Renal TMA was proven by kidney biopsy in 3 cases. We compared our patients with 4 patients who had a kidney biopsy for glomerular diseases (minimal change disease was used as a comparator because in this pathology there are usually no deposits of complement on the renal biopsy). By immunofluorescence, we found deposits of the membrane attack complex C5b9 along the glomerular and tubular membrane and also in the capillary wall in our patients as compared to control patients, suggesting the activation of complement cascade in this form of TMA (Fig. 1).

**Fig. 2:**
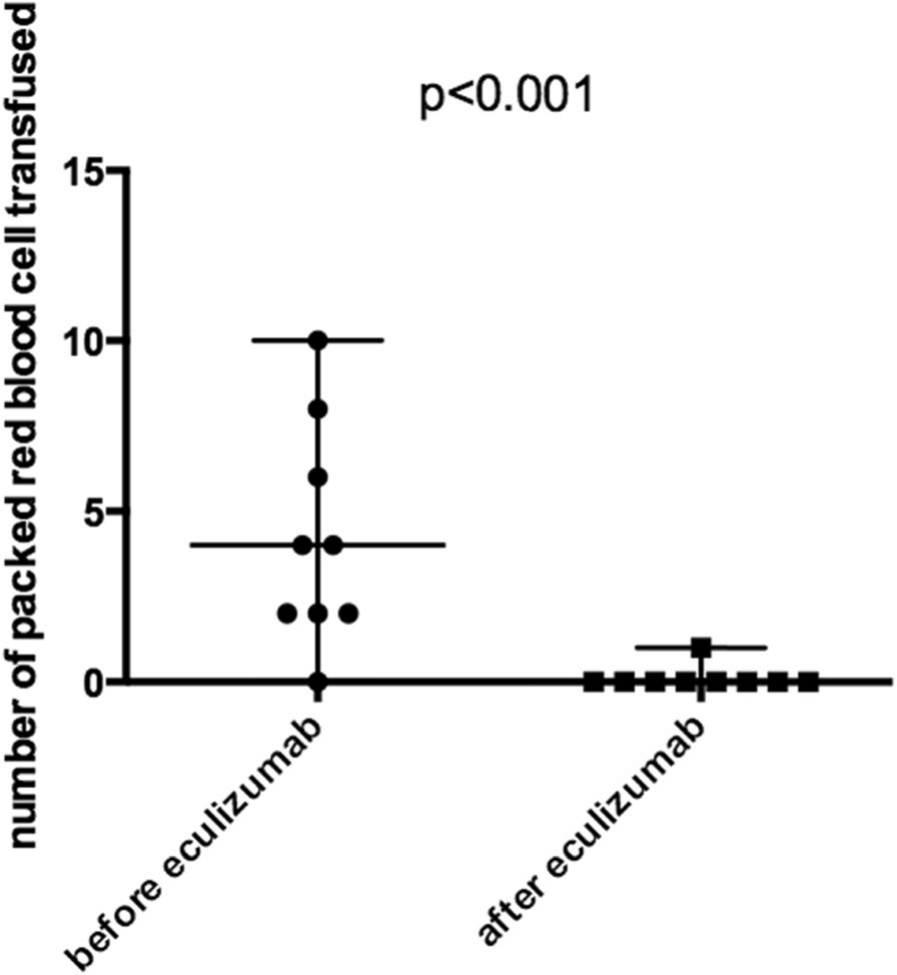
Comparison of packed red blood cell transfusion before and after eculizumab therapy. Quantitative values are expressed as median with range

All patients had their gemcitabine treatment stopped. First-line therapeutic plasma exchange (TPE) was performed in 5 patients (42%), with a median of 7 sessions (range 4–9) without significant benefit on hemolysis or renal function recovery. Eculizumab was started after a median delay of 15 days (range 4–44) following TMA diagnosis. A median of 4 injections (900 mg/injection, total 3600 mg) of eculizumab was administered (range 2–22). Of note, only three patients had received more than four injections of eculizumab. Hematological response was obtained in 10 patients (83%) and blood transfusion significantly decreased after the first infusion of eculizumab (median of 3 packed red blood cells (range 0–10) before treatment vs 0 (range 0–1) after one injection, *p* < 0.001) (Fig. 2). Two patients recovered renal function completely (17%), and 8 achieved a partial renal response (67%), with a median reduction of 8.5 mg/l of maximum creatinine level (range 2.5–47) (Table 1). After a median follow-up of 13 months, seven patients (58%) had persistent chronic renal failure with an estimated glomerular filtration rate (eGFR) below 60 ml/min/1.73m^2^. No treatment-associated adverse event was reported. Especially, no meningococcal infection was recorded during follow-up. No exacerbation or relapse of TMA were recorded after eculizumab discontinuation. Six patients (50%) died during follow-up, as an indirect complication of TMA with hemorrhagic shock (1 case) despite eculizumab treatment, or to cancer progression after a median of 9 months (range 2–13) following eculizumab initiation (5 cases). Six patients (50%) were in complete hematological response and at least partial renal response of TMA after eculizumab discontinuation allowing a switch to another antineoplastic agent (Table 1).

**Fig. 3:**
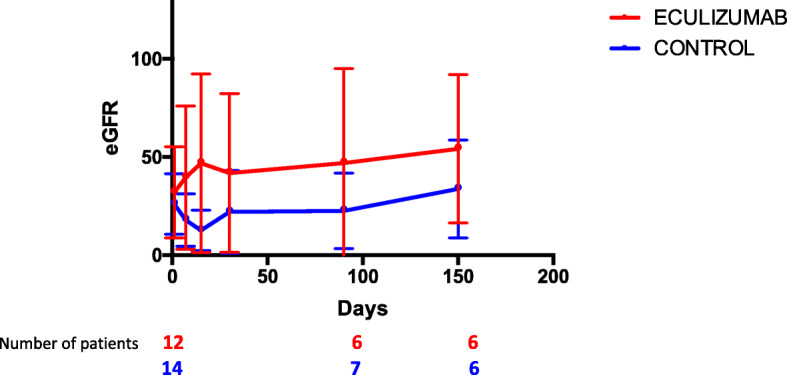
Evolution of renal function as a function of time in the eculizumab group and in the control group. Values expressed as mean and standard deviations

We compared patients with G-TMA treated with eculizumab with a control cohort of 14 patients who didn’t receive eculizumab treatment (Table 2). TPE were performed in 8 of them. Median baseline eGFR was comparable in the 2 groups, 95 (47–147) ml/min/1.73m^2^ in the control group and 106 (59–132) ml/min/1.73m^2^ in the eculizumab group. Compared to the control cohort, patients with G-TMA treated by eculizumab had a better renal outcome (Fig. 3). 83% of patients in eculizumab group had improvement of their renal function versus 64% in control group, and median eGFR was 45 (0–119) vs 33 (0–66) ml/min/1.73m^2^ respectively at the end of the follow up (Table 3). Of note, 2 patients (16%) still had end stage renal failure in the eculizumab group versus 3 patients (21%) in the control group.

**Table 2:** Characteristics of patients in the control group

Patient	Age (years old)	Type of cancer	Staging of AKI	Hemoglobin level (g/dl)	Platelets count (G/l)	LDH ratio (x normal)	Serum Creatinine level at diagnosis (mg/l)	Hematological response	Renal response	Outcome / Time to death or time of last follow-up for still alive patients (months)
1	56	Pancreatic, NA	3	12.3	79	1.9	44	Yes	Partial	Deceased / 72 m
2	33	Ovarian, M+	2	6.2	58	1.4	18	Yes	Partial	Deceased / 10 m
3	80	Pancreatic, M+	2	7.3	81	1.7	10	Yes	Complete	Deceased / 14 m
4	65	Pancreatic, M+	3	9.7	70	1.2	35	Yes	Partial	Deceased / 18 m
5	74	Pancreatic, M+	3, RRT	9.7	34	2.9	35	No	No	Alive / 3 m
6	66	Pulmonary, NA	3, RRT	4	146	3.9	94	Yes	No	Deceased / 1 m
7	59	Pancreatic, M+	3	6.9	85	1.5	41	Yes	Partial	Alive / 3 m
8	55	Pancreatic, M-	3	8.7	100	3.0	41	Yes	No	Deceased / 2 m
9	78	Pancreatic, M-	3	7.2	450	1.0	29	Yes	Complete	Alive / 24 m
10	56	Breast, M+	2	7.5	61	1.0	17	Yes	Complete	Alive / 2 m
11	58	Hepatic, NA	3, RRT	10.4	42	6.5	32.4	Yes	No	Deceased / 8 m
12	60	Pancreatic, M+	3	8.7	202	2.5	23	Yes	No	Deceased / 7 m
13	52	Hepatic, M+	3	9.1	96	5.8	50	Yes	Partial	Deceased / 10 m
14	73	Pancreatic, NA	3	9.9	430	3.1	29	Yes	Partial	Deceased / 14 m

*TMA* Thrombotic microangiopathy, *AKI* Acute kidney injury (AKI was assessed according to KDIGO classification 2012), *LDH* Lactate dehydrogenase, *RRT* Renal replacement therapy, *M-* No metastatic, *M+* Metastatic, *NA* Data not available

**Table 3:** Outcome of patients

	Eculizumab group *N* = 12 (%)	Control group *N* = 14 (%)
Renal response	10 (83)	9 (64)
Partial	8 (66)	6 (43)
complete	2 (17)	3 (21)
eGFR at onset (ml/min/1.73m^2)^	19 (0–76)	12 (0–31)
eGFR at the end of follow up	45 (0–119)	33 (0–66)

*eGFR* Estimated glomerular filtration rate. Quantitative values are expressed as median with range

## Discussion

We report here the largest case series of G-TMA treated by eculizumab. In our patients, we found that the transient use of eculizumab was efficient in controlling the hematologic disorders, by reducing significantly transfusion needs and by correcting thrombocytopenia. Remarkably, hematologic improvement was usually observed just after the two first injections of eculizumab, which strongly suggests a therapeutic action of eculizumab. However, we cannot rule out the hypothesis that the decrease in transfusion requirements was linked to the elimination of gemcitabine after discontinuation of this treatment. As in atypical HUS [14, 16], the use of eculizumab in G-TMA may improve renal function recovery. Indeed, 83% of patients in our study had a complete or partial renal remission within 2 to 4 weeks after complement blockade, suggesting again that eculizumab was efficient in controlling TMA. This two-step response with first a rapid improvement in cytopenias after the initiation of eculizumab followed by a more progressive renal improvement is reminiscent of the schedule of response observed in atypical HUS [14].

The pathophysiology of G-TMA is not well established. However, our data show C5b9 deposits in kidney biopsies supporting the hypothesis that the pathophysiology of G-TMA is at least partially related to complement activation, which may result from a direct endothelial toxicity of the drug. This transient complement activation seems to have no genetic background. A recent work has not revealed any pathogenic variant involved in the regulation of the alternate pathway of complement [17]. There still remains little evidence for quantitative analyses of complement proteins as valid biomarkers.

Reports of patients with G-TMA treated by eculizumab are rare [18–27]. To our knowledge, only 13 cases have been reported in literature, and a similar good outcome was observed. However, these results must be interpreted with caution due to publication bias. As opposed to the hematological response, renal remission is generally observed later and may occur after several weeks because of the process of endothelial healing. One could hypothesize that, similar to atypical HUS, an earlier initiation of eculizumab could allow a greater improvement in renal function recovery [14]. Moreover, there has been no exacerbation or relapse of TMA following eculizumab discontinuation, suggesting that a limited number of infusions may be sufficient to control the TMA process, in association with definitive gemcitabine withdrawal.

Although eculizumab raises cost concerns, these considerations should be weighted against a decreased burden of care, including lower transfusion needs, reduced needs for TPE, and possibly less renal replacement therapy with reduced length of hospitalisation in the intensive care unit during the early stages of AKI. As a result, this strategy could allow a significant improvement in patients’ quality of life, particularly when the underlying malignancy has a favourable prognosis.

Our results have the usual limitations of those of a retrospective study, in particular concerning the probable presence of confounding factors; moreover, the number of patients is relatively limited. Therefore, further larger controlled studies are needed to definitely confirm our results, which will be very difficult given the rarity of the disease. These studies should also address whether gemcitabine should be considered contraindicated after resolution of G-TMA. Only one patient had a genetic evaluation of the alternative complement pathway. Nevertheless, in France, a quantitative analysis of the complement is sometimes carried out in this context of TMAs secondary to gemcitabine. If this is abnormal, it is completed by the genetic evaluation. We now know that there is no pathogenic variant found in secondary TMAs in the vast majority of patients [17]. On the other hand, our study rather suggests a transient activation of the alternate pathway of complement. Finally, there were analyzable kidney biopsies in just 3 patients, so it is difficult to draw broad conclusions about the findings in G-TMA.

### Aknowledgements

The members of the Reference Centre for Thrombotic Microangiopathies (CNR-MAT) are: Azoulay Elie (Service de Réanimation Médicale, Hôpital Saint-Louis, Paris); Barbay Virginie (Laboratoire d’Hématologie, CHU Charles Nicolle, Rouen); Benhamou Ygal (Service de Médecine Interne, CHU Charles Nicolle, Rouen); Bordessoule Dominique (Service d’Hématologie, Hôpital Dupuytren, Limoges); Charasse Christophe (Service de Néphrologie, Centre Hospitalier de Saint-Brieuc); Chauveau Dominique (Département de Néphrologie et Transplantation d’Organes, CHU Rangueil, Toulouse); Choukroun Gabriel (Service de Néphrologie, Hôpital Sud, Amiens); Coindre Jean-Philippe (Service de Néphrologie, CH Le Mans); Coppo Paul (Service d’Hématologie, Hôpital Saint-Antoine, Paris); Corre Elise (Service d’Hématologie, Hôpital Saint-Antoine, Paris); Delmas Yahsou (Service de Néphrologie, Hôpital Pellegrin, Bordeaux); Deschenes Georges (Service de Néphrologie Pédiatrique, Hôpital Robert Debré, Paris); Devidas Alain (Service d’Hématologie, Hôpital Sud-Francilien, Corbeil-Essonnes); Fain Olivier (Service de Médecine Interne, Hôpital Saint-Antoine, Paris); Frémeaux-Bacchi Véronique (Laboratoire d’Immunologie, Hôpital Européen Georges Pompidou, Paris); Galicier Lionel (Service d’Immunopathologie, Hôpital Saint-Louis, Paris); Grange Steven (Service de Réanimation, CHU Charles Nicolle, Rouen); Guidet Bertrand (Service de Réanimation Médicale, Hôpital Saint-Antoine, Paris); Halimi Jean-Michel (Service de Néphrologie Pédiatrique, Hôpital Bretonneau, Tours); Hamidou Mohamed (Service de Médecine Interne, Hôtel-Dieu, Nantes); Herbrecht Raoul (service d’Oncologie et d’Hématologie, Hôpital de Hautepierre, Strasbourg); Jacobs Frédéric (Service de Réanimation Médicale, Hôpital Antoine Béclère, Clamart); Joly Bérangère (Service d’Hématologie Biologique, Hôpital Lariboisière, Paris); Kanouni Tarik (Unité d’Hémaphrèse, Service d’Hématologie, CHU de Montpellier); Lautrette Alexandre (Service de Néphrologie Pédiatrique B, Hôpital Hôtel-Dieu, Clermont-Ferrand); Le Guern Véronique (Unité d’Hémaphérèse, Service de Médecine Interne, Hôpital Cochin, Paris); Loirat Chantal (Service de Néphrologie Pédiatrique, Hôpital Robert Debré, Paris); Mira Jean-Paul (Service de Réanimation Médicale, Hôpital Cochin); Moulin Bruno (Service de Néphrologie, Hôpital Civil, Strasbourg); Mousson Christiane (Service de Néphrologie, CHU de Dijon); Ojeda Uribe Mario (Service d’Hématologie, Hôpital Emile Muller, Mulhouse); Ouchenir Abdelkader (Service de Réanimation, Hôpital Louis Pasteur, Le Coudray); Parquet Nathalie (Unité de Clinique Transfusionnelle, Hôpital Cochin, Paris); Peltier Julie (Urgences Néphrologiques et Transplantation Rénale, Hôpital Tenon, Paris); Perez Pierre (Service de Réanimation polyvalente, CHU de Nancy); Poullin Pascale (Service d’hémaphérèse et d’autotransfusion, Hôpital la Conception, Marseille); Pouteil-Noble Claire (Service de Néphrologie, CHU Lyon-Sud, Lyon); Presne Claire (Service de Néphrologie, Hôpital Nord, Amiens); Provôt François (Service de Néphrologie, Hôpital Albert Calmette, Lille); Ribeil Jean-Antoine (Service de Thérapie Cellulaire, Hôpital Necker-Enfants Malades, Paris); Rondeau Eric (Urgences Néphrologiques et Transplantation Rénale, Hôpital Tenon, Paris); Saheb Samir (Unité d’Hémaphérèse, Hôpital la Pitié-Salpétrière, Paris); Schlemmer Benoît (Service de Réanimation Médicale, Hôpital Saint-Louis, Paris); Seguin Amélie (Service de Réanimation Médicale, CHU de Caen); Stépanian Alain (Service d’Hématologie Biologique, Hôpital Lariboisière, Paris); Vernant Jean-Paul (Service d’Hématologie, Hôpital la Pitié-Salpétrière, Paris); Veyradier Agnès (Service d’Hématologie Biologique, Hôpital Lariboisière, Paris); Vigneau Cécile (Service de Néphrologie, Hôpital Pontchaillou, Rennes); Wynckel Alain (Service de Néphrologie, Hôpital Maison Blanche, Reims); Zunic Patricia (Service d’Hématologie, Groupe Hospitalier Sud-Réunion, la Réunion).

## Supplementary Information

**Additional file 1.**

## Data Availability

The datasets used and/or analysed during the current study are available from the corresponding author on reasonable request.

## Abbreviations

AKI: Acute Kidney Injury
eGFR: Estimated Glomerular Filtration Rate
G-TMA: Gemcitabine-induced Thrombotic Microangiopathy
HUS: Hemolytic Uremic Syndrome
LDH: Lactate Dehydrogenase
RRT: Renal Replacement Therapy
TMA: Thrombotic Microangiopathy
TPE: Therapeutic Plasma Exchange
TTP: Thrombocytopenic Thrombotic Purpura
