# Supplementary material for: Eculizumab in gemcitabine-induced thrombotic microangiopathy: experience of the French thrombotic microangiopathies reference centre
Source: BMC Nephrol. 2021 Jul 21;22:267. doi: 10.1186/s12882-021-02470-3 (PMC8293501; doi:10.1186/s12882-021-02470-3)
Supplement: Supplementary file 1 — Additional file 1. [file 12882_2021_2470_MOESM1_ESM.docx]

**Supplemental Data:**

| Patients of eculizumab group | Analysis of Complement pathway |
| --- | --- |
| 1 | Normal plasma level of C3, C4, CH50 |
| 2 | Decreased plasma level of C3, C4, CH50  Normal expression of CD46 |
| 3 | Normal plasma level of C3, C4, CH50, Factor H and Factor I  No anti-factor H antibodies |
| 4 | Normal plasma level of C3, C4, CH50, Factor H and Factor I  Normal expression of CD46  No anti-factor H antibodies |
| 5 | Normal plasma level of C3, C4, CH50, Factor H and Factor I  Normal expression of CD46  No anti-factor H antibodies |
| 6 | Normal plasma level of C3, C4, CH50, Factor H and Factor I  Normal expression of CD46  No anti-factor H antibodies |
| 7 | Normal plasma level of C3, C4, CH50 |
| 8 | Normal plasma level of C3, C4, CH50 |
| 9 | No data |
| 10 | Decreased plasma level of C3  Normal plasma level of C4 and CH50 |
| 11 | Normal plasma level of C3, C4, CH50, Factor H and Factor I  Normal expression of CD46  No anti-factor H antibodies |
| 12 | Normal plasma level of C3, C4, CH50 |
| Patients of control group |  |
| 1 | Normal plasma level of C3, C4, CH50 |
| 2 | Normal plasma level of C3, C4, CH50 |
| 3 | No data |
| 4 | No data |
| 5 | Normal plasma level of C3, C4, CH50 |
| 6 | Normal plasma level of C3, C4, CH50 |
| 7 | Normal plasma level of C3, C4, CH50 |
| 8 | Normal plasma level of C3, C4, CH50 |
| 9 | Decreased plasma level of C4 and CH50  Normal plasma level of Factor H and Factor I  Normal expression of CD46  No anti-factor H antibodies |
| 10 | No data |
| 11 | No data |
| 12 | No data |
| 13 | No data |
| 14 | No data |
